# Memantine shifts cancer cell metabolism via AMPK1/2 mediated energetic switch in A549 lung cancer cells

**DOI:** 10.17179/excli2020-2890

**Published:** 2021-02-04

**Authors:** Gulsah Albayrak, Funda Demirtas Korkmaz

**Affiliations:** 1 Department of Medical Biology, Faculty of Medicine, Ufuk University, Ankara, Turkey; 2 Department of Medical Biology, Faculty of Medicine, Giresun University, Giresun, Turkey

**Keywords:** memantine, lung cancer, drug re-purposing, cancer cell metabolism

## Abstract

Memantine is used to prevent glutamate-mediated excitotoxicity and neurodegeneration in Alzheimer's disease. As glutamine is one of the major source of anabolism in fast growing cancer cells, we aimed to interfere with the cancer cell metabolism in A549 lung cancer cells by using memantine. The effects of memantine on cell cycle progression and cell death in A549 cells were assessed by MTT assay and PI staining. Cells were treated with 0.25 mM memantine for 48 hours and then cell metabolism (*AMPKA1*, *AMPKA2*, *HIF1A*, *B-catenin*, *PKM*), apoptosis (*p53*, *p21*, *Bax*, *Bcl-XL*, *NOXA*, *PUMA*) and autophagy related (*LC3B-I*, *LC3B-II*, *SQSTM1*) mRNA and protein expressions were investigated by RT-qPCR and western blotting. Memantine decreased cell viability significantly in a concentration-dependent manner by inducing G0/G1 cell cycle arrest. Our results suggest that memantine activates AMPK1/2 significantly (p=0.039 and p=0.0105) that led cells through apoptosis and autophagy by decreasing cancer cell metabolism regulators like HIF1A, B-catenin and PKM as the consequence of this energetic shift. Memantine represents a useful tool to target metabolism in cancer cells. Therefore, it might be used a new repurposed drug in cancer treatment.

## Introduction

Metabolism is one of the hallmarks of cancer as cancer cells undergo metabolic reprogramming to support their accelerated anabolic growth (Ward and Thompson, 2012[20]). Tumorigenesis is dependent on cellular metabolism due to the consequence of direct or indirect oncogenic mutations (Pavlova and Thompson, 2016[15]). Therefore, tumor cell metabolism is thought as cancers Achilles' Heel (Kroemer and Pouyssegur, 2008[7]). Targeting altered energy metabolism hold great promise for therapeutic intervention in cancer (Zhang and Yang, 2013[21]).

Repurposing old drugs, other than their original indication is increasing as drug discovery process is time consuming and expensive (Pushpakom et al., 2018[16]). Large number of compounds enter into phase I trials whereas there is a high risk of failure especially in cancer drugs due to the lack of efficacy and toxicity related concerns (Beijersbergen, 2020[2]). 

Memantine is used to prevent excess glutamate toxicity in Alzheimer's disease. There are on-going clinical trials about its potential use in preventing the cognitive dysfunctions (Brown et al., 2013[3]) or its use in combination with the other chemotherapy drugs for cancer treatment (Maraka et al., 2019[13]). Memantine is able to cross the blood brain barrier and has half life of 60-100 hours where 80 % of the drug circulates without any change in its chemical properties (Keiski, 2017[6]). Recently, memantine has been found to exert anti-cancer activity in cancer cells (Lowinus et al., 2019[11]). Memantine's anti-cancer activity is not only limited to the brain area but also for other cancer types. In this study we aimed to target cancer cell metabolism by using memantine in A549 lung cancer cells. Thefore we assessed its effect on cell cycle progression and cellular death by MTT assay and PI staining. Then we investigated cell metabolism related targets *AMPKA1*, *AMPKA2*, *HIF1A*, *B-catenin*, *PKM*, apoptosis related *p53*, *p21*, *Bax*, *Bcl-XL*, *NOXA*, *PUMA* and autophagy related *LC3B-I*, *LC3B-II*, *SQSTM1 *mRNA and protein expressions in order to gain broader insight into the anticancer mechanism of action for this drug.

## Materials and Methods

### Cell culture and chemicals

A549 cells were grown in DMEM medium supplemented with 10 % Fetal bovine serum (FBS) (Sigma-Aldrich, St Louis, MO, USA). Cells were grown in 5 % CO_2_ at 37 °C. Memantine was supplied from Sigma-Aldrich, St Louis, MO, USA. Cells were treated with 0.125-4 mM memantine for 24 and 48 hours. Memantine was dissolved in sterile, non-pyrogenic distilled water.

### Cellular cytotoxicity assay

3-(4,5-dimethylthiazol-2-yl)-2,5-diphenyl-tetrazolium bromide (MTT) assay was used to evaluate the cytotoxic effect of memantine. Cells were seeded into the 96 well plate and cultured for overnight. A549 cells were treated with memantine for 24 and 48 hours. MTT solution (5 mg/ml in PBS) was added to each well after treatment and the plate was incubated for 4 h at 37 °C. DMSO was added to solubilize the formazan crystals and the plate was further incubated at 37 °C for 30 min. Absorbance ratio was measured by SpectraMax M3 (Molecular Devices, USA) microplate reader at 570 nm.

### Protein expression profiles by Western blot

After 48 h treatment of A549 cells with memantine, cells were washed with PBS and scraped into the RIPA lysis buffer containing 1mM PMSF followed by sonication for 15 seconds. Samples were centrifuged for 15 minutes at 14000 rpm at 4 °C and the supernatant was collected. Proteins were quantified by using the BCA Assay Kit (Thermo Pierce, Rockford, IL, USA). Protein lysates (20 μg) were heated for 5 minutes at 95 °C in LDS non-reducing sample buffer (Pierce, Rockford, IL, USA) and then loaded to the 10 % Tris-glycin gels. The gels were transferred to the PVDF membrane (Merck Millipore, Darmtadt, Germany) at 300 mAmp for 90 minutes. Membranes were blocked with 5 % non-fat milk powder in TBS-T for 1 hour at room temperature and incubated overnight at 4 °C with the primary antibodies for HIF1A, B-catenin, total PKM, B-actin, LC3B and SQSTM1 at 1:1000 dilution (Thermo Pierce, Rockford, IL, USA). Blots were washed with TBS-T subsequently. Protein bands were detected by using the secondary antibody (Thermo Pierce, Rockford, IL, USA) and the blots were visualized by BioVision ECL Western Blotting Substrate Kit (Biovision, California, USA).

### Cell cycle analysis by propidium iodide assay

A549 cells were harvested and centrifuged for 5 min at 1500 rpm. Cell pellets were fixed with 1 ml 70 % ice-cold ethanol and incubated at -20 °C overnight. Cells were washed in 6ml PBS, stained for 30 min with 20 ng/ml Propidium Iodide Solution and RNAase (Thermo Fischer Scientific). Cell cycle analysis was performed in FACSCalibur flow cytometer (BD Bioscience) and plotted in Graphpad prism software.

### Gene expression analysis by RT-qPCR

RNA was isolated by using Trizol reagent (Invitrogen, Thermo Fischer Scientific, USA) 2000 ng total RNA was reverse transcribed by Superscript III cDNA Synthesis Kit (Invitrogen, Thermo Fischer Scientific, USA). Forward and reverse primer sequences were provided in Table 1(Tab. 1). 10-20 cycles of specific target amplification was performed by SYBR Green qPCR master mix. RT- qPCR analysis was performed in Roche LightCycler qPCR. Gapdh was used as internal control when calculating Cq value. ΔΔCq method was used to quantify the gene expression levels. Data was analyzed and plotted in Graphpad prism software.

### Statistical analysis

Experiments were done in biological and technical triplicates. Statistical analysis was performed by using GraphPad Prism software. Differences between the control and treatment groups were analyzed by using the t-test. The results were expressed as the mean ± standard error mean. Kaplan Meier analysis of overall survival data for high and low HIF1A expression in patients (Cut off high 50 % and Cut off low 50 %). The graph was plotted by using GEPIA (Gene Expression Profiling Interactive Analysis).

## Results

### Memantine decreased cell viability significantly via inducing cell cycle arrest at G0/G1 phase

**Figure 1:**
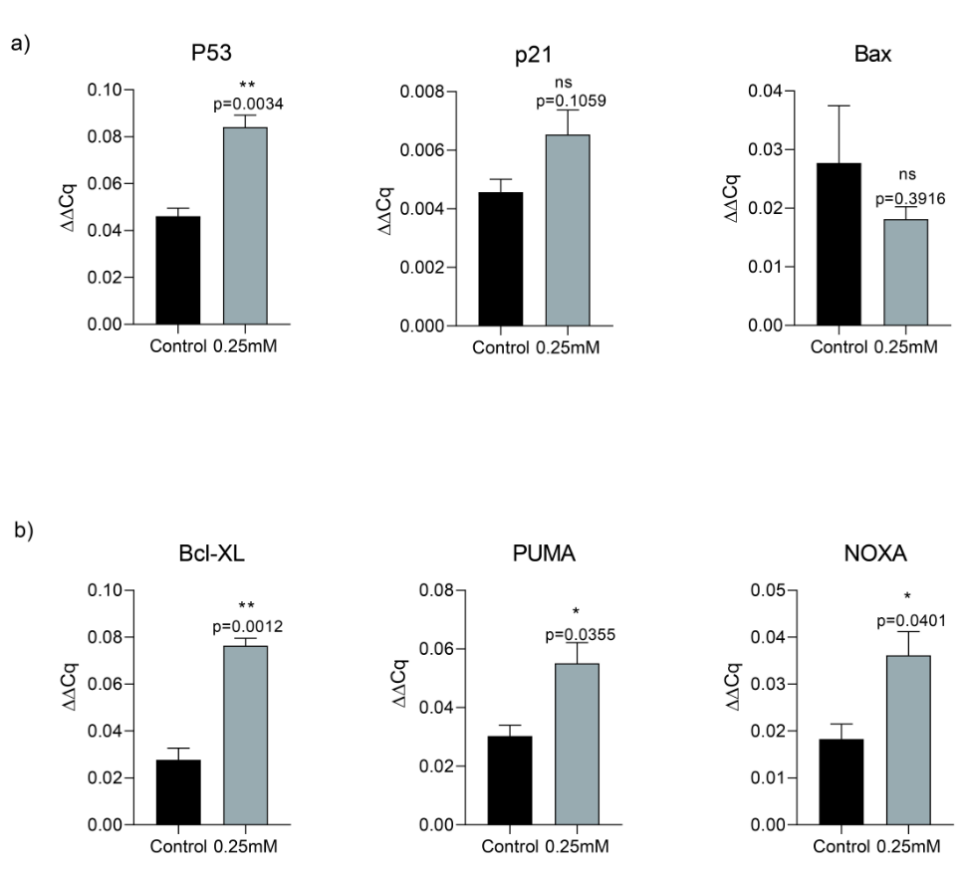
a) Memantine's effect on A549 cell viability at 24 and 48 hours with different memantine concentrations (0.125-4 mM). Memantine decreased A549 lung cancer cell viability significantly (p=0.0094, two-tailed paired t-test) in MTT assay. b) Effect on cell cycle progression after 0.25 mM memantine treatment in Propidium Iodide assay. Control cells had 0.1 % Sub G1, 62.8 % G0/G1, 9.9 % S and 21.3 % G2/M phase whereas 0.25 mM memantine treated cells had 0.6 % Sub G1, 82.7 % G0/G1, 3.6 % S and 10.8 % G2/M phase. Schematic representation of cell cycle phases after memantine treatment. Each experiment was performed in triplicate (n=3).

Memantine's effect on A549 cell viability was investigated at 24 and 48 hours with different memantine concentrations (0.125-4 mM). Memantine decreased cell viability significantly (p=0.0094, two-tailed paired t-test) in MTT assay for both 24 and 48 hours in a dose-dependent manner (Figure 1a(Fig. 1)). At 0.25 mM concentration cell viability was found as 76.58 % for 48 hours. Therefore those concentrations were chosen for further analysis to investigate the molecular mechanism of this anticancer activity. Memantine's effect on cell cycle profile was assessed by propidium iodide staining by flow cytometry. A549 cells were treated with 0.25 mM of memantine for 48 hours and cell cycle profiles were compared with the untreated control cells. Control cells had 0.1 % Sub G1, 62.8 % G0/G1, 9.9 % S and 21.3 % of the cells at G2/M phase whereas memantine treated cells had 0.6 % Sub G1, 82.7 % G0/G1, 3.6 % S and 10.8 % of the cells at G2/M phase. Corresponding cell cycle phases were represented as pie chart graphs in Figure 1b(Fig. 1). Memantine triggered the accumulation of the cells at G0/G1 phase at 48 hours.

### Memantine activated AMPK1/2 and decreased HIF1A, B-catenin, total PKM protein expressions

**Figure 2:**
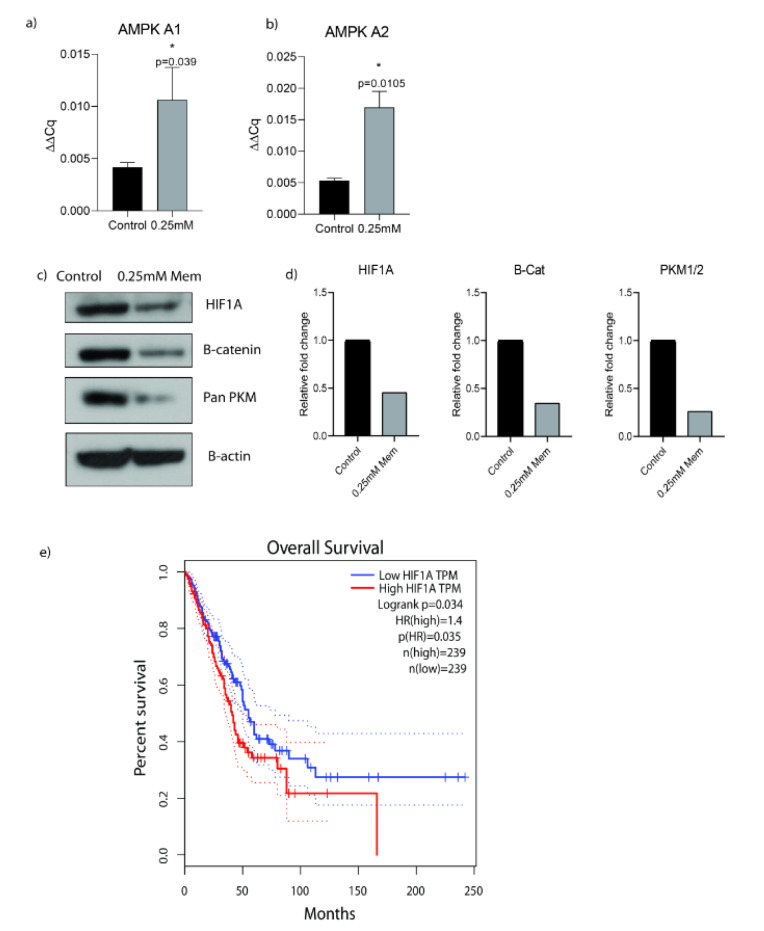
a) Memantine's effect on the gene expression analysis of AMPK A1 after 48 h 0.25 mM treatment in A549 lung cancer cells. b) Memantine's effect on the gene expression analysis of AMPK A2 after 48h 0.25mM treatment in A549 lung cancer cells. c) Protein expressions of HIF1A, B-Catenin and PKM after 48 h 0.25 mM treatment in A549 lung cancer cells. B-actin was used as loading control. PKM antibody detects endogenous levels of total PKM (including M1 and M2) protein. Representative blots were shown as n=3. d) ImageJ analysis was done and protein expression levels were represented as relative fold changes. e) Kaplan-Meier survival analysis of HIF1A gene with median-centered high vs low expression (Cut off high 50% and Cut off low 50%) (hazard ratio = 1.4 n = 239, p=0.035). The graph was plotted by using GEPIA (Gene Expression Profiling Interactive Analysis). Each experiment was performed in triplicate (n=3).

We investigated AMPK A1 and AMPK A2 mRNA expression levels after 0.25 mM memantine treatment for 48 h in A549 cells in order to assess memantine's effect on cancer cell metabolism. Memantine activated AMPK A1 and AMPK A2 genes at mRNA level significantly (p=0.039 and p=0.0105) (Figure 2a, 2b(Fig. 2)). Then we checked the potential downstream targets of AMPK activated oncogenic pathway by looking at HIF1A, B-catenin and total PKM protein expression levels. Memantine treatment decreased HIF1A, B-catenin and PKM protein expression levels (Figure 2c, 2d(Fig. 2)). All of these targets are involved in cancer cell metabolism related changes in oncogenic transformation of the cells. Hypoxia-inducible factor 1 (HIF-1) is thought as the master regulator of cancer metabolism by activating glucolytic metabolism (Semenza, 2010[17]). We compared the overall survival of high and low HIF1A expression by Kaplan-Meier survival analysis by using publicly available data set GEPIA. In LUAD (The Cancer Genome Atlas Lung Adenocarcinoma) samples, higher HIF1A expression is found as an indicator of lower overall survival rates (Figure 2e(Fig. 2)) (n = 239, p=0.035).

### Memantine induced cell death via p53 activation that directed cells into the apoptosis and autophagy

**Figure 3:**
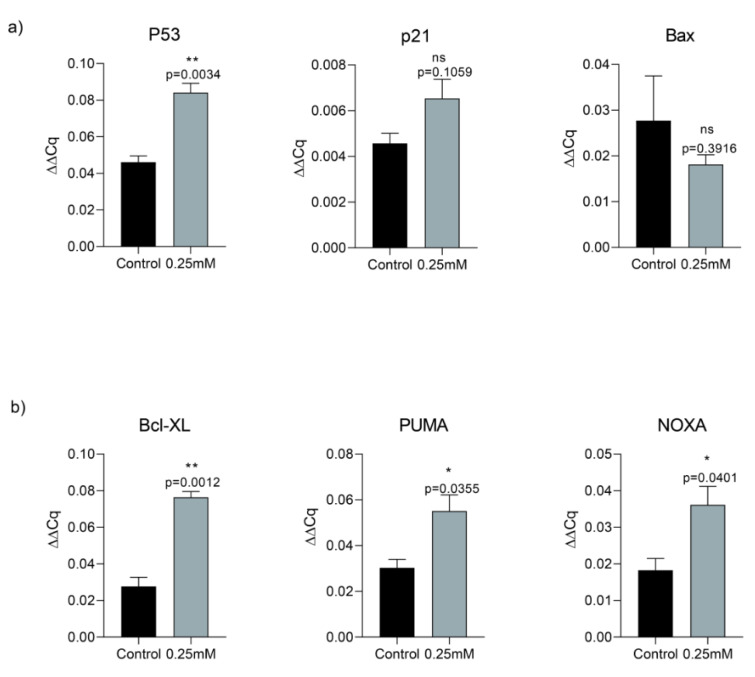
a) Memantine's effect on the gene expression analysis of P53, P21 and Bax after 48 h 0.25 mM treatment in A549 lung cancer cells. b) Memantine's effect on the gene expression analysis of Bcl-XL, PUMA, NOXA after 48 h 0.25 mM treatment in A549 lung cancer cells. Gene expressions were normalized to the Gapdh levels. Error bars were represented as SEM. Each experiment was performed in triplicate (n=3).

In order to assess memantine's effect on cell death, we investigated its role on p53-dependent pathway of apoptosis. A549 cells were treated with 0.25 mM memantine for 48 h. p53, p21, Bax, Bcl-XL, PUMA and NOXA mRNA expression levels were measured by qRT-PCR. Memantine increased p53 level significantly (p=0.034). Memantine also increased p21 mRNA expression level (Figure 3a(Fig. 3)). In addition to that memantine increased pro-apoptotic gene expression of Bcl-XL, PUMA, NOXA levels significantly (Figure 3b(Fig. 3)). We found that as memantine activated p53 mediated apoptosis, downstream pathway of apoptotic genes are also activated (Bcl-XL, PUMA, NOXA). PUMA is known as “p53 upregulated modulator of apoptosis”. Therefore our results were consistent with the potential activation of P53-dependent pathway of apoptosis.

**Figure 4:**
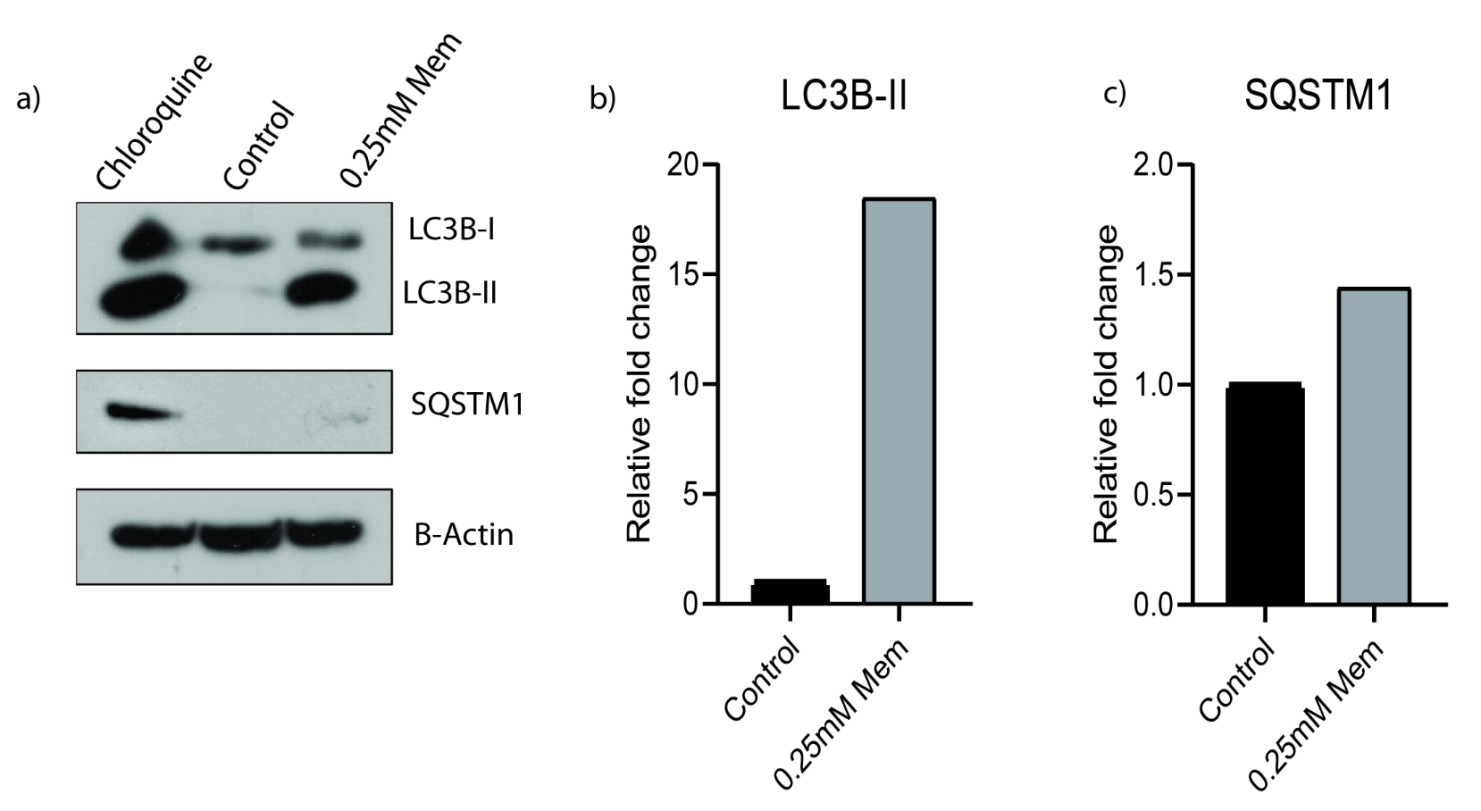
a) Memantine’s effect on the protein expressions of LC3B-I, LC3B-II and SQSTM1 after 48 h 0.25 mM treatment in A549 lung cancer cells. A549 cells were treated with10 µM Chloroquine for 48 h and it was used as a positive control. b) LC3B-II and c) SQSTM1 protein expressions are the indicators of autophagy. B-actin was used as loading control. Representative blots were shown as n=3.

Then we further investigated memantine's effect on autophagy. We checked the autophagy related protein expression levels of LC3B-I, LC3B-II and SQSTM1 expressions by western blot. Presence of LC3B in autophagosomes and the conversion of LC3 to the lower migrating form as LC3-II is an indicator of autophagy. Memantine increased LC3B-II protein expression after 48 h 0.25 mM memantine treatment (Figure 4a(Fig. 4)). A549 cells were treated with 10 μM Chloroquine for 48 h and this was used as a positive control to compare the induction of autophagy. Memantine also increased SQSTM1 protein expression but this change was much lower than LC3B-II induction (Figure 4a, 4c(Fig. 4)).

## Discussion

Cancer cells benefit the advantage of rapid glycolysis process during their metabolism even when oxygen is available. This phenomena of cancer cells known as the Warburg effect (Liberti et al., 2016[10]). Cancer cells undergo metabolic reprogramming that contributes the oncogenic transformation. Therefore their metabolic liabilities could be exploited to treat cancer (Luengo et al., 2017[12]).

Drug repurposing studies are gaining more interest as it enables shorter routes to clinic with the use of de-risked compounds, with potentially lower development costs and shorter development timelines (Pushpakom et al., 2018[16]). In this study, we aimed to investigate Alzheimer' drug memantine as a potential cell metabolism regulator in cancer treatment. Therefore we evaluated a wide range of cell cycle relevant changes upon memantine treatment in A549 lung cancer cells.

AMPK activation leads to the activation of p53 that induces a decrease in cell cycle progression at cellular level (Andrzejewski et al., 2018[1]). Our results indicate that Memantine triggered AMPK activation that shifted the cancer cell metabolism. That shift in cell metabolism downregulated HIF1A, B-catenin and total PKM protein levels (including M1 and M2). Essential requirements for cancer cell metabolism are high glucose consumption and lactate production.

Pyruvate kinase (PK), which catalyzes the final step of glycolysis, has emerged as a potential regulator of this metabolic phenotype (Chaneton and Gottlieb, 2012[4]). 

Previous studies have shown the importance of HIF1A and PKM as single bio-markers for predicting prognosis and metastasis in NSCLC (non-small cell lung cancer) (Lai et al., 2020[8]). Recently HIF-1a was found to act as a metabolic switch between glycolytic driven and oxidative phosphorylation driven T-regs in glioblastoma (Miska et al., 2019[14]).

Memantine altered lung cancer cell metabolism by regulating AMPK1/2 levels. AMPK is a cellular energy sensor that is conserved in all eukaryotic cells (Garcia and Shaw, 2017[5]). This effect might activate p53 response and direct cells into apoptosis by increasing apoptotic gene expressions (Bcl-XL, Puma, Noxa). AMPK promotes ATP conservation under metabolic stress conditions by activating catabolic metabolism pathways such as autophagy (Tamargo-Gómez and Mariño, 2018[18]). Our results have shown that AMPK activation and as a consequence of that stress conditions; memantine induced G0/G1 cell cycle arrest that directed cells into the apoptosis and autophagy. Wnt-B-catenin pathway has important role in the regulation of cellular metabolism as upregulated WNT pathway shifts mitochondrial oxidative phosphorylation towards to the Warburg glycolysis metabolism in favor of cancer cells (Lecarpentier et al., 2019[9]). Memantine reduced B-catenin protein expression levels that indicates its role in this pathway as well.

One recent study shows that lung cancer cells are dependent on glycolysis like many other cancer cells in order to boost their anabolic demand. Therefore inhibition of anaerobic glycolysis might be a promising target for the targeted therapy (Vanhove et al., 2019[19]). Providing that memantine decreased the master oncogenic regulators like HIF1A, B-catenin and PKM, our results conclude that memantine could be used in targeting cancer cell metabolism.

## Conflict of interest

The authors declare that they have no conflict of interest. 

## Funding

This research received no specific grant from any funding agency.

## Ethical approval

This article does not contain any studies with human participants or animals performed by any of the authors. 

## Figures and Tables

**Table 1:**
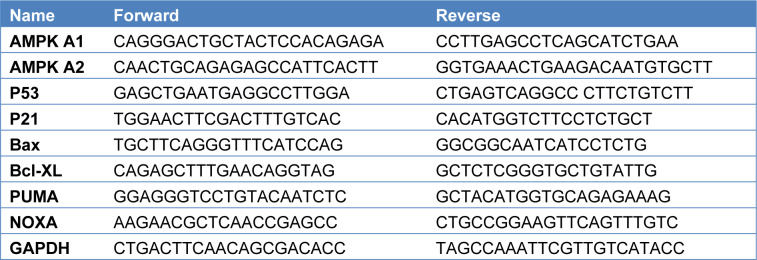
RT-PCR Primer list

## References

[1] Andrzejewski S, Siegel PM, St-Pierre J (2018). Metabolic profiles associated with metformin efficacy in cancer. Front Endocrinol.

[2] Beijersbergen RL (2020). Old drugs with new tricks. Nature Cancer.

[3] Brown PD, Pugh S, Laack NN, Wefel JS, Khuntia D, Meyers C (2013). Memantine for the prevention of cognitive dysfunction in patients receiving whole-brain radiotherapy: A randomized, double-blind, placebo-controlled trial. Neuro Oncol.

[4] Chaneton B, Gottlieb E (2012). Rocking cell metabolism: Revised functions of the key glycolytic regulator PKM2 in cancer. Trends Biochem Sci.

[5] Garcia D, Shaw RJ (2017). AMPK: Mechanisms of cellular energy sensing and restoration of metabolic balance. Mol Cell.

[6] Keiski, MA, Heidenreich K (2017). Memantine: A safe and tolerable NMDA antagonist with potential benefits in traumatic brain injury. New therapeutics for traumatic brain injury.

[7] Kroemer G, Pouyssegur J (2008). Tumor cell metabolism: Cancer's achilles' heel. Cancer Cell.

[8] Lai YH, Chen WN, Hsu TC, Lin C, Tsao Y, Wu S (2020). Overall survival prediction of non-small cell lung cancer by integrating microarray and clinical data with deep learning. Sci Rep.

[9] Lecarpentier Y, Schussler O, Hébert JL, Vallée A (2019). Multiple targets of the canonical WNT/β-catenin signaling in cancers. Front Oncol.

[10] Liberti MV, Locasale JW (2016). The Warburg effect: How does it benefit cancer cells?. Trends Biochem Sci.

[11] Lowinus T, Heidel FH, Bose T, Nimmagadda SC, Schnöder T, Cammann C (2019). Memantine potentiates cytarabine-induced cell death of acute leukemia correlating with inhibition of Kv1.3 potassium channels, AKT and ERK1/2 signaling. Cell Commun Signal.

[12] Luengo A, Gui DY, Vander Heiden MG (2017). Targeting metabolism for cancer therapy. Cell Chem Biol.

[13] Maraka S, Groves MD, Mammoser AG, Melguizo-Gavilanes I, Conrad CA, Tremont-Lukats IW (2019). Phase 1 lead-in to a phase 2 factorial study of temozolomide plus memantine, mefloquine, and metformin as postradiation adjuvant therapy for newly diagnosed glioblastoma. Cancer.

[14] Miska J, Lee-Chang C, Rashidi A, Muroski ME, Chang AL, Lopez-Rosas A (2019). HIF-1α is a metabolic switch between glycolytic-driven migration and oxidative phosphorylation-driven immunosuppression of tregs in glioblastoma. Cell Rep.

[15] Pavlova NN, Thompson CB (2016). The emerging hallmarks of cancer metabolism. Cell Metab.

[16] Pushpakom S, Iorio F, Eyers PA, Escott KJ, Hopper S, Wells A (2018). Drug repurposing: progress, challenges and recommendations. Nat Rev Drug Discov.

[17] Semenza GL (2010). HIF-1: Upstream and downstream of cancer metabolism. Curr Opin Genet Dev.

[18] Tamargo-Gómez I, Mariño G (2018). AMPK: Regulation of metabolic dynamics in the context of autophagy. Int J Mol Sci.

[19] Vanhove K, Graulus GJ, Mesotten L, Thomeer M, Derveaux E, Noben JP (2019). The metabolic landscape of lung cancer: New insights in a disturbed glucose metabolism. Front Oncol.

[20] Ward PS, Thompson CB (2012). Metabolic reprogramming: A cancer hallmark even Warburg did not anticipate. Cancer Cell.

[21] Zhang Y, Yang JM (2013). Altered energy metabolism in cancer: A unique opportunity for therapeutic intervention. Cancer Biol Ther.

